# Motion Correction in High‐Resolution 3D Brain MRSI Without Water and Lipid Suppression

**DOI:** 10.1002/mrm.70128

**Published:** 2025-10-10

**Authors:** Ziwen Ke, Yibo Zhao, Rong Guo, Yudu Li, Wen Jin, Huixiang Zhuang, Yao Li, Zhi‐Pei Liang

**Affiliations:** ^1^ Beckman Institute for Advanced Science and Technology University of Illinois at Urbana‐Champaign Urbana Illinois USA; ^2^ National Engineering Research Center of Advanced Magnetic Resonance Technologies for Diagnosis and Therapy, School of Biomedical Engineering Shanghai Jiao Tong University Shanghai China; ^3^ Siemens Medical Solutions USA, Inc. St. Louis Missouri USA; ^4^ Department of Bioengineering University of Illinois at Urbana‐Champaign Urbana Illinois USA; ^5^ Department of Electrical and Computer Engineering University of Illinois at Urbana‐Champaign Urbana Illinois USA

**Keywords:** constrained reconstruction, non‐water‐suppressed MRSI, retrospective motion correction, sensitivity encoding

## Abstract

**Purpose:**

To develop an effective method for correcting head motion in high‐resolution, non‐water‐suppressed MRSI.

**Methods:**

MRSI scans are susceptible to subject motion due to the long data acquisition time required for sufficient spatial‐spectral encodings. The problem is more serious in non‐water‐suppressed MRSI experiments since motion artifacts in the water and lipid signals make their removal even more challenging. To address this problem, we propose a novel motion correction method that detects and discards motion‐corrupted k‐space data using TR‐wise linear navigators. The discarded data are replaced with reconstructed data obtained by reformulating motion correction as a missing data reconstruction problem using sensitivity encodings. Extrinsic priors for water and lipid signals and intrinsic priors for metabolite signals are incorporated in the motion correction process. We evaluated its performance on a spectroscopic phantom and three human groups: (1) five healthy adults performing three different voluntary motion patterns; (2) a healthy child; and (3) a cerebral hemorrhage patient with involuntary movements.

**Results:**

In phantom and healthy subjects, the proposed method produced high‐quality water images and metabolite maps that closely matched motion‐free references, with 10%–20% quantitative gains in image/map quality (higher PSNR/SSIM, lower NRMSE). In the child and patient data, motion artifacts were noticeably reduced, with 20%–30% reductions in NAA linewidth and fitting error in the child, and ˜50% reductions in coefficient‐of‐variation in the patient.

**Conclusion:**

An effective motion‐correction technique has been developed for high‐resolution non‐water‐suppressed MRSI. This method has the potential to significantly enhance the robustness and clinical applicability of non‐water‐suppressed MRSI.

## 1 Introduction

Head motion poses a major challenge in MRSI brain scans due to the long data acquisition time [1, 2]. Motion artifacts can severely degrade image quality, resulting in lipid contamination and spectral distortions [3, 4, 5]. This problem becomes more pronounced in non‐water‐suppressed MRSI [6, 7, 8, 9, 10, 11, 12, 13, 14, 15, 16, 17] because unsuppressed water and lipid signals are three to four orders of magnitude stronger than metabolite signals. Any motion artifacts in the reconstructed water and lipid signals will affect their removal, thus resulting in errors in metabolite signal estimation [18, 19, 20].

Several motion detection and correction methods have been developed for MRI/MRSI. For motion detection, state‐of‐the‐art approaches include optical tracking and navigator‐based methods. Optical tracking uses camera‐based systems to detect head movements [21, 22, 23], requiring no modifications to MR pulse sequences but specialized equipment. Navigator‐based methods integrate additional navigators into pulse sequences to track motion, including cloverleaf [24], orbital/spherical [25, 26], spiral [27], and volumetric image navigators [28, 29, 30, 31]. While these methods require no extra hardware, additional excitations/readouts for navigators may increase overall scan time. For motion correction, existing methods typically estimate motion parameters first and then use them to correct motion effects. These methods can be broadly categorized into prospective and retrospective methods. Prospective methods [27, 28, 29, 30, 31, 32] integrate real‐time motion tracking and correction during image acquisition, using techniques such as extended Kalman filter [27], low‐power gradient modulated localization [28, 29, 32], estimated phase difference mapping [30], and registered localization [31]. Retrospective techniques [33, 34, 35] estimate motion parameters post‐acquisition and apply corrections during image reconstruction, using techniques like entropy of spatial gradient [33], motion matrix inversion [34], or deep learning [35]. Both prospective and retrospective methods rely on accurate motion parameter estimation [36]. This is particularly challenging in MRSI scans due to several factors: (1) Conventional MR navigators are unsuitable for water‐suppressed MRSI due to significantly reduced SNR. One solution is to acquire navigators within the water suppression module [32]. While this allows TR‐wise motion estimation, the navigator module requires > 500 ms per TR, making it impractical for short‐TR FID‐MRSI (e.g., TR < 300 ms). (2) Even with accurate motion parameter estimation, retrospective motion correction is difficult to achieve, as existing forward model‐based methods are often ill‐posed. (3) Head movements disrupt the steady state of transverse magnetizations, leading to signal fluctuations and inconsistencies for different TRs. These challenges lead to insufficient motion correction, a significant concern in non‐water‐suppressed MRSI, where motion‐induced water residuals can greatly degrade spectral quantification accuracy.

We introduce a practical motion correction method specifically designed for the high‐resolution, non‐water‐suppressed MRSI technique known as SPICE (SPectroscopic Imaging by exploiting spatiospectral CorrElation) [6, 7, 8, 9, 10, 11, 12, 13, 14, 15, 16, 17]. Instead of estimating motion parameters, our method focuses on motion detection, synergistically integrating ultrafast motion navigators in data acquisition with constrained image reconstruction to effectively remove motion artifacts in non‐water‐suppressed MRSI. The efficacy of our method has been validated through phantom and human experiments.

## 2 Methods

Motion artifacts arise from local k‐space inconsistencies caused by head movements [37], leading to both spatial and spectral distortions. In this paper, we propose a three‐step strategy for motion correction: (1) identifying motion‐corrupted k‐space locations, which separate the k‐space data into several motion‐free blocks, (2) estimating and correcting the phase inconsistencies between these k‐space blocks, and (3) replacing the motion‐corrupted k‐space data with reconstructed data obtained using constrained reconstruction. Since motion detection is a significantly easier problem than motion parameter estimation, it can be achieved with efficient TR‐wise linear navigators. These TR‐wise navigators enable the detection of subject motion in high temporal resolution, thus minimizing the amount of data to be discarded. Afterward, we estimated and corrected the phase inconsistencies between different k‐space blocks. After identifying the motion‐corrupted k‐space locations and correcting inconsistencies in the remaining data, the motion correction problem was formulated as a constrained image reconstruction problem, synergistically integrating coil sensitivity encoding and prior image constraints to effectively re‐generate the motion‐corrupted data. The constrained reconstruction problem was solved separately for the water/lipid signals and metabolite signals so that stronger priors could be imposed on the water/lipid signals without impacting the reconstruction of the metabolite signals.

### 2.1 Data Acquisition

**FIGURE 1:**
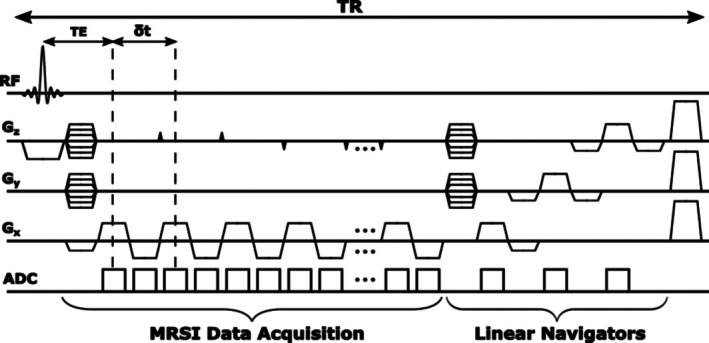
Pulse sequence of high‐resolution non‐water‐suppressed MRSI (SPICE) with TR‐wise motion navigators. Navigator signals are acquired at the end of each TR in the form of orthogonal linear navigators for each axis. The readout gradients of the navigators share the same shape parameters as the MRSI readout gradient, and the total duration for acquiring this set of navigators is ∼5 ms.

The proposed data acquisition scheme is an extension of the SPICE sequence, as depicted in Figure 1. Compared with conventional MRSI pulse sequences, the SPICE sequence has several key features: (1) Short TR of 160 ms, achieving high imaging speed with efficient SNR; (2) Ultrashort TE of 1.6 ms, leading to reduced T2 signal decay and thereby improving SNR; (3) No water or lipid suppression, reducing scan time while leveraging unsuppressed water and lipid signals for field mapping, field drift monitoring, and motion tracking; (4) Extended EPSI trajectory, achieving large k‐space coverage for high spatial resolution; (5) Fully sampled k‐space data, preserving metabolite SNR and improving tolerance to motion corruption.

To enable motion detection, we inserted a set of linear navigators at the end of each TR. Specifically, after the last pair of bipolar EPSI trajectories, we applied rewinder gradients in the *y* and *z* directions to remove the phase encoding effects. We then acquired three navigators along the *x*, *y*, and *z* directions, respectively, to capture any motion observable as translations in each direction. The total acquisition time for these navigators was ˜5 ms. Despite being acquired at the end of each TR, the navigators have sufficient SNR due to the short TR (160 ms) and no water suppression used in the SPICE sequence. Notably, navigator acquisition neither extends the total scan time nor disrupts the established magnetization steady state, as the excitation pulses and TR values remain unchanged compared to the original SPICE sequence.

### 2.2 Data Processing

#### 2.2.1 Motion Detection

We chose a particular TR during the scanning interval (e.g., the first one) as the reference point. Any head motion occurring between this reference TR and a subsequent TR will affect the “consistency” of the navigators, making it detectable. In each TR, the proposed sequence acquired three linear navigators. A Fourier transform was first applied to each of the navigators to generate 1D projection profiles of the subject along the *x*, *y*, and *z* directions. Motion‐induced positional shifts were then identified by comparing these projections with the reference projections. In our implementation, linear translations along the *x*, *y*, and *z* directions (denoted as Δx[n], Δy[n], and Δz[n], respectively) were determined from the L_2_ difference and cross‐correlation metrics.

Although the motion parameters estimated from the linear navigators cannot fully characterize head motion (e.g., rotational movements are ignored), they effectively detect head motion occurring at specific TRs. The overall translational displacement within the *n* ^th^ TR was calculated as $\Delta d\left[n\right]=\sqrt{\Delta x{\left[n\right]}^{2}+\Delta y{\left[n\right]}^{2}+\Delta z{\left[n\right]}^{2}}$, which reliably indicates motion occurrence (Figure S1). To improve the robustness against measurement noise, these displacements were denoised using a sliding window average, before calculating Δd[n].

Instead of using a predefined threshold to identify motion, which is sensitive to subject‐dependent signal scaling variations, we introduced a locally adaptive method. Specifically, a moved standard deviation (moved SD) transformation was applied to the translational displacement $\sigma \left[n\right]=\sqrt{\frac{1}{W}\sum_{w=n-W/2+1}^{n+W/2}{\left(\Delta d\left[k\right]-\mu \left[n\right]\right)}^{2}}$, where W is the time window and μ[n] is the mean signal within the window. We selected a time window to W=12, as it effectively balanced the temporal resolution of motion detection and robustness to scaling variations. A self‐adaptive threshold was then determined as the mean of the moved SD, effectively capturing TRs affected by head motion.

#### 2.2.2 Motion Correction

**FIGURE 2:**
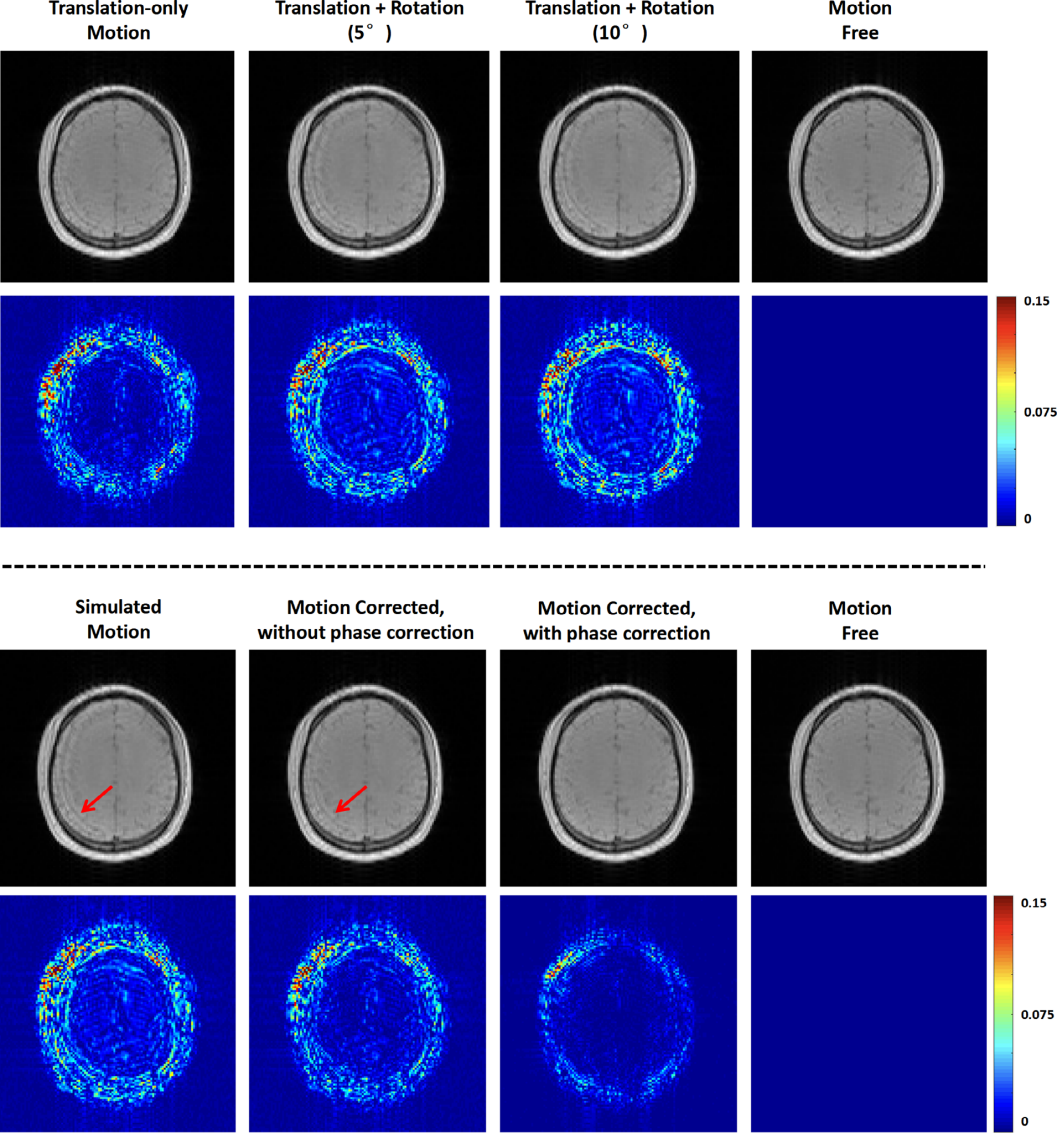
Motion effects and the benefit of phase correction. Top: Left to right: Simulated motion with translation only; translation + rotation (5°); translation + rotation (10°); motion‐free reference. Magnitude (top row) and difference maps to the reference (bottom row) show that translation causes most of the motion errors, while rotations add only a small amount. This motivates focusing on correcting translation. Bottom: Results for “translation + rotation (5°)” case. Left to right: Simulated motion, motion correction without phase correction, motion correction with phase correction, motion‐free reference. With phase correction, motion errors are significantly removed, and the result is closer to the reference.

With the motion identified, the k‐space data were segmented into several blocks: within each block, there were minimal motion effects; between blocks, there could be motion‐induced inconsistencies, as the subject may not return to the original position after movement. These inconsistencies, if not corrected well, would lead to image artifacts [22]. In this study, we observed that such inconsistencies mainly exhibit themselves as phase errors, as illustrated in Figure 2. Based on this observation, we developed a phase correction scheme to handle the data inconsistency issue caused by no‐return movements. In this scheme, we estimated linear phase inconsistencies between different k‐space blocks using an iterative algorithm, starting with the estimations from navigator signals as an initial guess, then updating the estimation based on measured k‐space data. More specifically, we first estimated the translations between TRs before and after the motion from navigator signals, as described in Section 2.2.1. These translations were then corrected from the k‐space data, and we used SENSE‐based reconstruction [38] (details described below in Equation 5) to obtain a reference image ${\hat{\rho }}^{\left(0\right)}$. Afterward, we re‐estimated the phase inconsistencies between each k‐space block ${\left\{d_{c}\right\}}_{c=1}^{N_{c}}$ and the k‐space data corresponding to the SENSE‐reconstructed reference image ${\left\{{\hat{d}}_{c}^{\left(0\right)}\right\}}_{c=1}^{N_{c}}≔{\left\{ℱ\left(S_{c}⊙{\hat{\rho }}^{\left(0\right)}\right)\right\}}_{c=1}^{N_{c}}$:

(1) $$\left\{{\hat{a}}_{x,i},{\hat{a}}_{y,i},{\hat{a}}_{z,i}\right\}=\underset{\left\{a_{x,i},a_{y,i},a_{z,i}\right\}}{\arg\;\min}\sum_{c=1}^{N_{c}}{\left\|\Omega_{i}\left(∠{\hat{d}}_{c}^{\left(0\right)}-∠A\left(a_{x,i},a_{y,i},a_{z,i}\right)d_{c}\right)\right\|}_{2}^{2},$$

where ${\left\{\Omega_{i}\right\}}_{i=1}^{N}$ denotes the sampling operator corresponding to the i ^th^ k‐space block; ∠· denotes the phase operator for complex data, and $A\left(a_{x,i},a_{y,i},a_{z,i}\right)$ the operator applying linear phase $e^{a_{x,i}k_{x}+a_{y,i}k_{y}+a_{z,i}k_{z}}$ to k‐space data. With $\left\{{\hat{a}}_{x,i},{\hat{a}}_{y,i},{\hat{a}}_{z,i}\right\}$ determined, $d_{c}$ was then updated as follows:

(2) $${\tilde{d}}_{c}^{\left(0\right)}=\sum_{i=1}^{N}\Omega_{i}A\left({\hat{a}}_{x,i},{\hat{a}}_{y,i},{\hat{a}}_{z,i}\right)d_{c},$$

With the updated and more consistent k‐space data, the SENSE reconstruction was performed again to yield better reconstruction results for reference. Then the phase correction (Equations 1 and 2) was updated based on the new reference for better phase estimation. These steps were performed iteratively until convergence (changes in estimated parameters < 0.2 mm).

After the motion‐corrupted k‐space locations were identified and inconsistencies between k‐space blocks were sufficiently corrected, the next step was to recover the discarded motion‐corrupted data. This was formulated as a reconstruction problem to obtain spatiotemporal signal ρ(x,t) from undersampled data:

(3) $$\begin{array}{cc} & \text{Given}:d_{c}\left(k,t\right)=\int S_{c}\left(x\right)⊙\rho \left(x,t\right)e^{-i2\pi k\cdot x}dx+\varepsilon_{c}\left(k,t\right),\\ & \;\text{for}\;k\;\mathrm{at}\;\text{motion‐free locations}\\ & \text{Determine}:\rho \left(x,t\right) \end{array}$$

where $d_{c}\left(k,t\right)$ denotes the uncorrupted k‐space data acquired from the c ^th^ receiving coil, $S_{c}\left(x\right)$ denotes the sensitivity profiles, and $\varepsilon_{c}\left(k,t\right)$ the measurement noise. This formulation differs from standard motion correction schemes that explicitly incorporate motion parameters into the forward model of image reconstruction, requiring accurate yet challenging motion parameter estimation [36]. Instead, we discarded the motion‐corrupted data and re‐generated the missing data using constrained image reconstruction, eliminating the need for exact motion parameters.

We solved the reconstruction problem in Equation (3) separately for water/lipid signals and metabolite signals to allow the incorporation of strong intrinsic and/or extrinsic priors on the water/lipid signals without biasing the metabolite signal reconstruction. In this work, we used total variation penalty as the intrinsic priors to enforce spatial smoothness, and motion‐free reference image from other scans or subjects as the extrinsic priors, which served as a spatial and spectral constraint during final reconstruction. Both intrinsic and extrinsic priors were applied in reconstructing the water/lipid signals, while only intrinsic priors (spatial smoothness constraints) were used for metabolite signals.

More specifically, for water and lipid reconstruction, we assumed that a motion‐free reference signal was available. This assumption is realistic since many motion‐free SPICE brain datasets are available (from the same subject in longitudinal studies or from a different subject). Note that priors for both lipid and water signals were incorporated in the reference to mitigate motion artifacts and reduce their contaminations in metabolite maps. The reference water and lipid data were first registered to the new experimental data using nonlinear deformation. The generalized series (GS) model [39] was then used to compensate for the discrepancy between the registered reference data (denoted as $\rho_{\mathrm{ref}}\left(x,t\right)$) and the new experimental data:

(4) $$\rho \left(x,t\right)=\rho_{\mathrm{ref}}\left(x,t\right)\cdot \sum_{l=-L/2}^{L/2}b_{l}\left(t\right)e^{i2\pi l△k\cdot x}$$

where $\left\{b_{l}\left(t\right)\right\}$ are the GS coefficients, and L is the GS order. The GS model efficiently captures prior information in the reference image, leading to fewer degrees of freedom than the conventional Fourier series model [39]. Its optimality has been justified previously under the minimal cross‐entropy principle [40]. With the registered reference image and GS model, the motion‐corrected water and lipid signals, $\rho_{\mathrm{wl}}\left(x,t\right)$, were reconstructed by solving the following optimization problem:

(5) $$\begin{array}{ll} {\hat{\rho }}_{\mathrm{wl}},{\hat{b}}_{l} & =\arg\min_{\rho_{\mathrm{wl}},b_{l}}\sum_{c=1}^{N_{c}}{\left\|d_{c}-\Omega ℱ\left(S_{c}⊙G\left(\rho_{\mathrm{ref}}\right)\;b_{l}\right)\right\|}_{2}^{2}\\ & \;+\lambda {\left\|\mathrm{TV}\left(\rho_{\mathrm{wl}}\right)\right\|}_{2}^{2},\text{ s}.t.\;\rho_{\mathrm{wl}}=G\left(\rho_{\mathrm{ref}}\right)\;b_{l} \end{array}$$

where Ω is the motion sampling operator, ℱ the Fourier transform operator, TV the total variation operator, and G the GS model operator. This problem was effectively solved using the alternating minimization (AM) algorithm [41]. After motion correction of the water/lipid signals, nuisance removal was performed following the SPICE processing pipeline [6, 7, 8, 9, 10, 11, 12, 13, 14, 15, 16, 17].

After removal of the water/lipid signals, the reconstruction problem in Equation (1) was addressed for metabolite signals by solving the following optimization problem:

(6) $${\hat{\rho }}_{m}=\arg\min_{\rho_{m}}\sum_{c=1}^{N_{c}}{\left\|d_{c}^{\mathrm{nr}}-\Omega ℱ\left(S_{c}⊙\rho_{m}\right)\right\|}_{2}^{2}+\lambda {\left\|\mathrm{TV}\left(\rho_{m}\right)\right\|}_{2}^{2},$$

where $d_{c}^{\mathrm{nr}}$ denotes the uncorrupted k‐space data after nuisance removal, and $\rho_{m}$ is the metabolite signals to be reconstructed. This problem incorporated only mild intrinsic priors, that is, sensitivity encoding [38] and smoothness, to avoid undesired estimation bias. Equation (6) is a convex optimization problem, and was effectively solved using the PCG algorithm [42]. After reconstruction, motion‐corrupted k‐space was re‐generated. The motion‐corrected data (with water and lipid signal removed) were then processed using the existing SPICE data processing pipeline [12] to generate the desired metabolite maps.

### 2.3 Experimental Setup

Experimental data were acquired using the 3T Siemens Prisma (for phantom and healthy subject data) and Biograph mMR scanners (for patient data).

For phantom experiments, a spectroscopic phantom was used, consisting of a cylindrical water jar containing nine glass vials filled with brain metabolite solutions at physiological concentrations. To simulate motion, the phantom was placed in three different positions, and raw data from these three individual scans were merged in k‐space to produce motion‐corrupted data. The scan protocols included a commercial MPRAGE scan (FOV = 240 × 240 × 192 mm^3^, matrix size = 256 × 256 × 192, TE = 2.29 ms, TI = 900 ms, TR = 1900 ms, 4.6 min), three consecutive SPICE scans (FOV = 240 × 240 × 72 mm^3^, matrix size = 110 × 78 × 24, TE = 1.6 ms, TR = 160 ms, echo‐spacing = 1.76 ms, flip angle = 27°, 8 min), and a fast FLASH scan for sensitivity map estimation (FOV = 240 × 240 × 72 mm^3^, matrix size = 128 × 128 × 24, TE = 2.0 ms, TR = 4.6 ms, flip angle = 12°, 20 s).

For in vivo experiments, five healthy volunteers were scanned with approval from the IRB of the University of Illinois. Each volunteer underwent four SPICE scans: in one scan, they were instructed to remain still, serving as the motion‐free reference. In the other three scans, they were instructed to perform voluntary head movements following three distinct patterns representative of practical motion. Specifically, for all scenarios, the volunteers were instructed to move at around 1.25 and 3.75 min during the scan. In Scenario (1), the volunteers were asked to perform short movements and to return to the original position. In Scenario (2), the volunteers were asked to perform short movements but to move to another position. In Scenario (3), the volunteers were asked to perform a long and continuous movement. To evaluate performance under real‐world clinical conditions, involuntary motion data were also acquired from a 10‐year‐old healthy child at Shanghai Children's Medical Center and a patient with cerebral hemorrhage at Huashan Hospital, both with IRB approval.

Quantitative evaluation was performed to assess image quality. When motion‐free reference data were available, metrics including PSNR, SSIM, and NRMSE were computed. In the absence of ground truth, the coefficient of variation (CoV) [43] was used to assess the spatial stability of the reconstructed images, and full width at half maximum (FWHM) and fitting error [44] were used to evaluate spectral quality. CoV is defined as the ratio of the standard deviation to the mean of voxel intensities within the motion‐affected white‐matter region. Lower CoV indicates a more homogeneous signal and fewer motion‐induced artifacts. FWHM and fitting error were derived from a parametric spectral model similar to LCModel [12, 45]: from the fitted spectra, NAA FWHM and the fitting error were estimated following [30], where the fitting error was defined as the Euclidean norm of the residual between the measured and fitted spectra, normalized by the norm of the original signal.

## 3 Results

**FIGURE 3:**
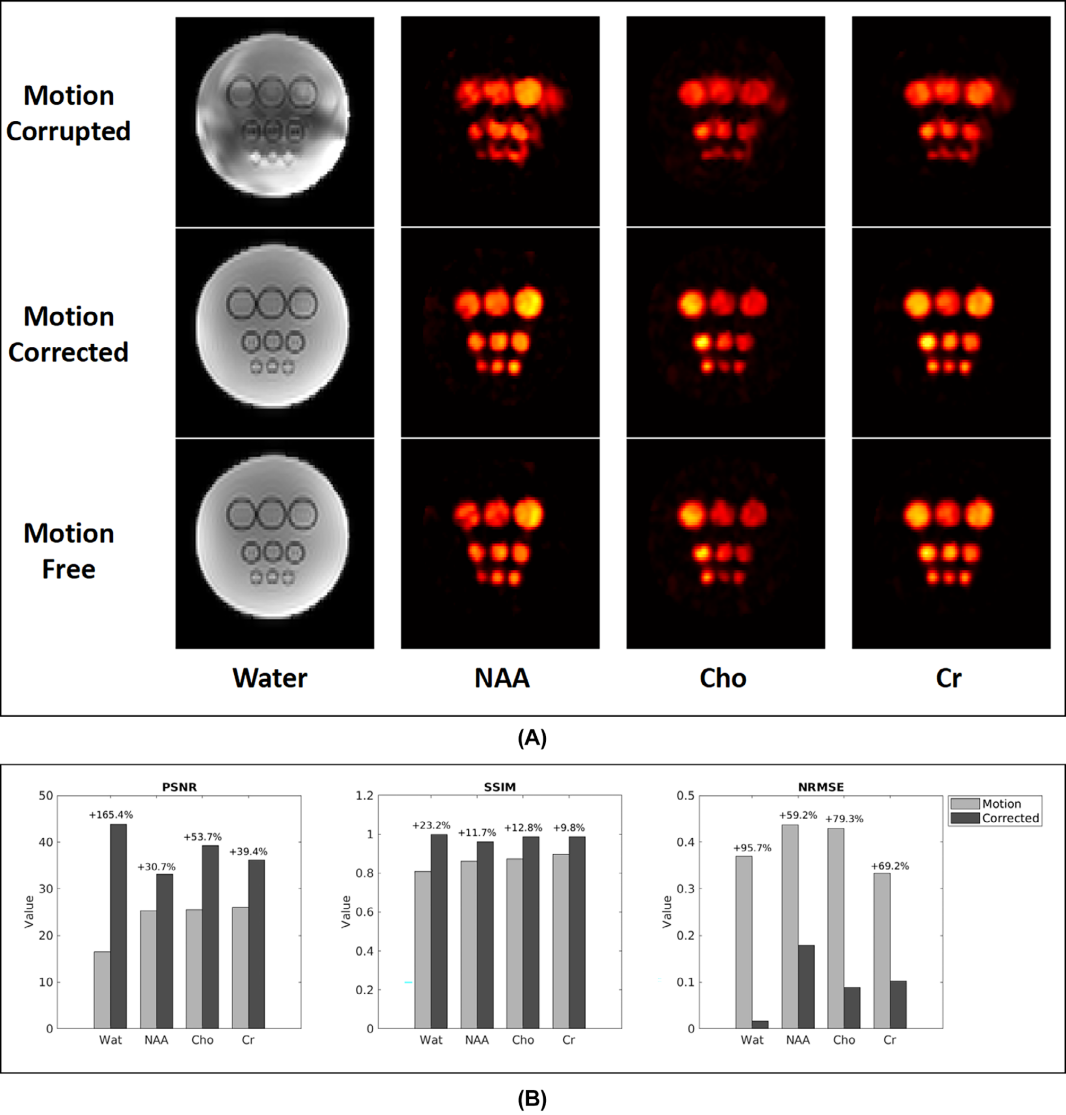
Comparison of phantom results obtained from the motion‐corrupted data, motion‐corrected data, and motion‐free data. (A) Water and metabolite (NAA, Cho, and Cr) images. (B) Quantitative evaluation using PSNR, SSIM, and NRMSE. The proposed motion correction method effectively reduces motion‐induced artifacts in both water and metabolite images, as evidenced by improved image quality and quantitative metrics.

Figure 3 shows the reconstructed water and metabolite (NAA, Cho, and Cr) images from the phantom data. In the motion‐corrupted data, substantial distortions were evident in both images. The proposed method effectively corrected these artifacts, yielding results that closely matched those from the benchmark phantom data. Quantitatively, the correction led to improvements of 30%–165% in PSNR, 10%–20% in SSIM, and 60%–90% reductions in NRMSE.

**FIGURE 4:**
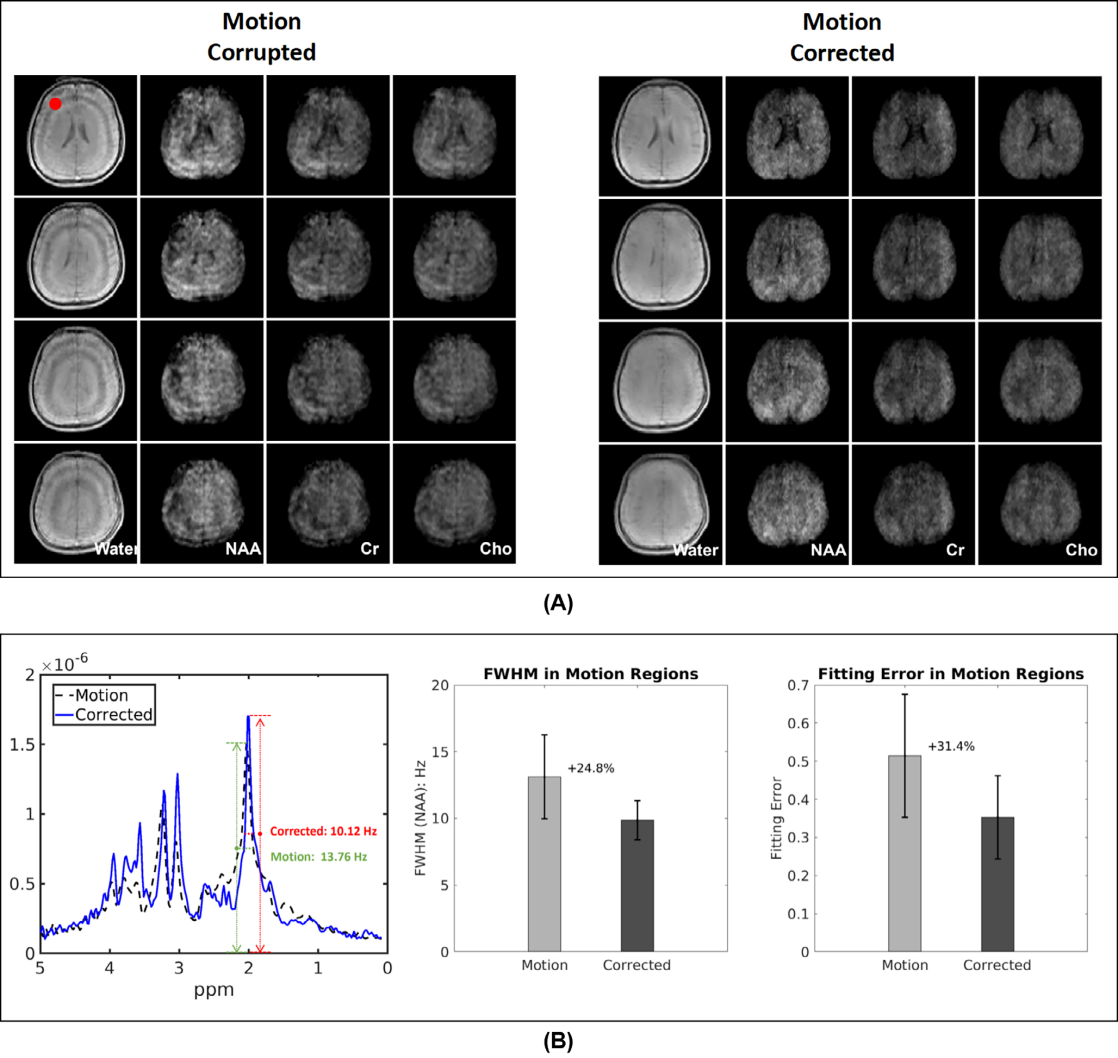
Comparison of water images and metabolite maps of a healthy 10‐year‐old child obtained from the motion corrupted data and motion‐corrected data. (A) Water and metabolite (NAA, Cho, and Cr) images reconstructed from motion‐corrupted and motion‐corrected data. (B) Quantitative evaluation using NAA FWHM and fitting error. The proposed motion correction method effectively reduces motion artifacts and enhances image quality across all metabolite maps.

Figure 4 presents motion correction results for a 10‐year‐old child. Significant motion artifacts were observed in both the water and metabolite maps. In particular, the water image exhibited prominent ring artifacts, while the metabolite maps showed irregular signal distributions. After motion correction, the prominent rings visible in the water image were eliminated, and the metabolite maps better matched the expected distributions in normal subjects. Quantitatively, FWHM improved by 25% and fitting error improved by 30%.

**FIGURE 5:**
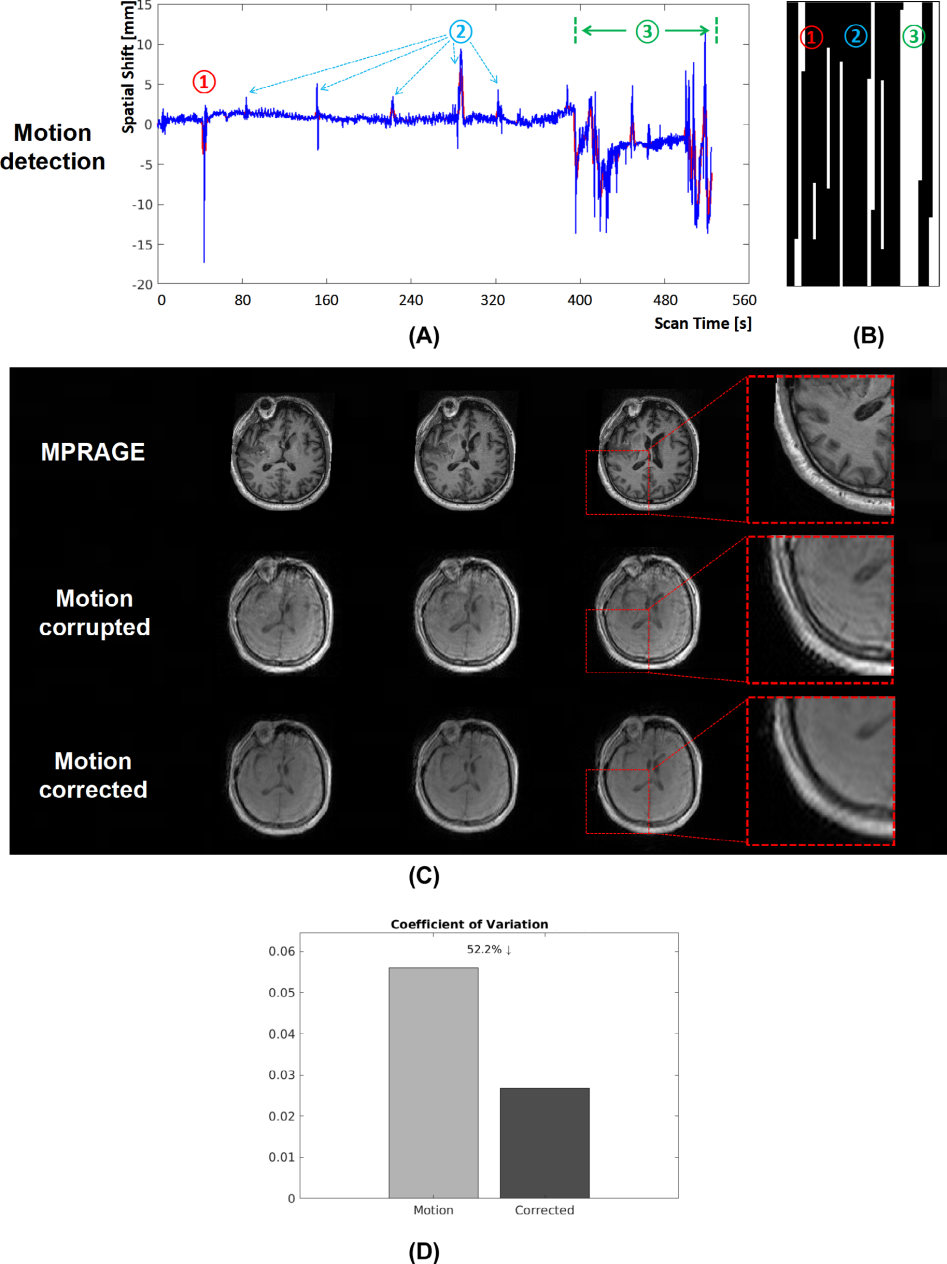
Motion detection and correction for a patient diagnosed with cerebral hemorrhage. (A) The estimated translation of a specific direction, extracted from the navigator signal (blue line: the original profile; red line: the denoised profile). (B) The detected motion mask, indicating the locations where motion occured within the k‐space. (C) The motion corrupted image and its corresponding motion corrected version. Red boxes: zoom‐in views. (D) Quantitative evaluation using CoV.

Figure 5 shows the results for a patient with cerebral hemorrhage in a clinical setting. In Figure 5a, the projection profile of a specific direction is displayed, where the noisy profiles (blue line) and the denoised profiles (red line) reveal significant motion events at the marked Points 1, 2, and 3. Figure 5b shows the detected motion‐corrupted k‐space locations derived from these projection profiles. Figure 5c presents the motion‐corrupted images, and the corresponding motion‐corrected results. The original image exhibited prominent motion artifacts that severely degraded image quality, which were effectively removed using the proposed method, substantially improving the overall image quality. Quantitatively, CoV improved by ˜52.2%, as shown in Figure 5d.

**FIGURE 6:**
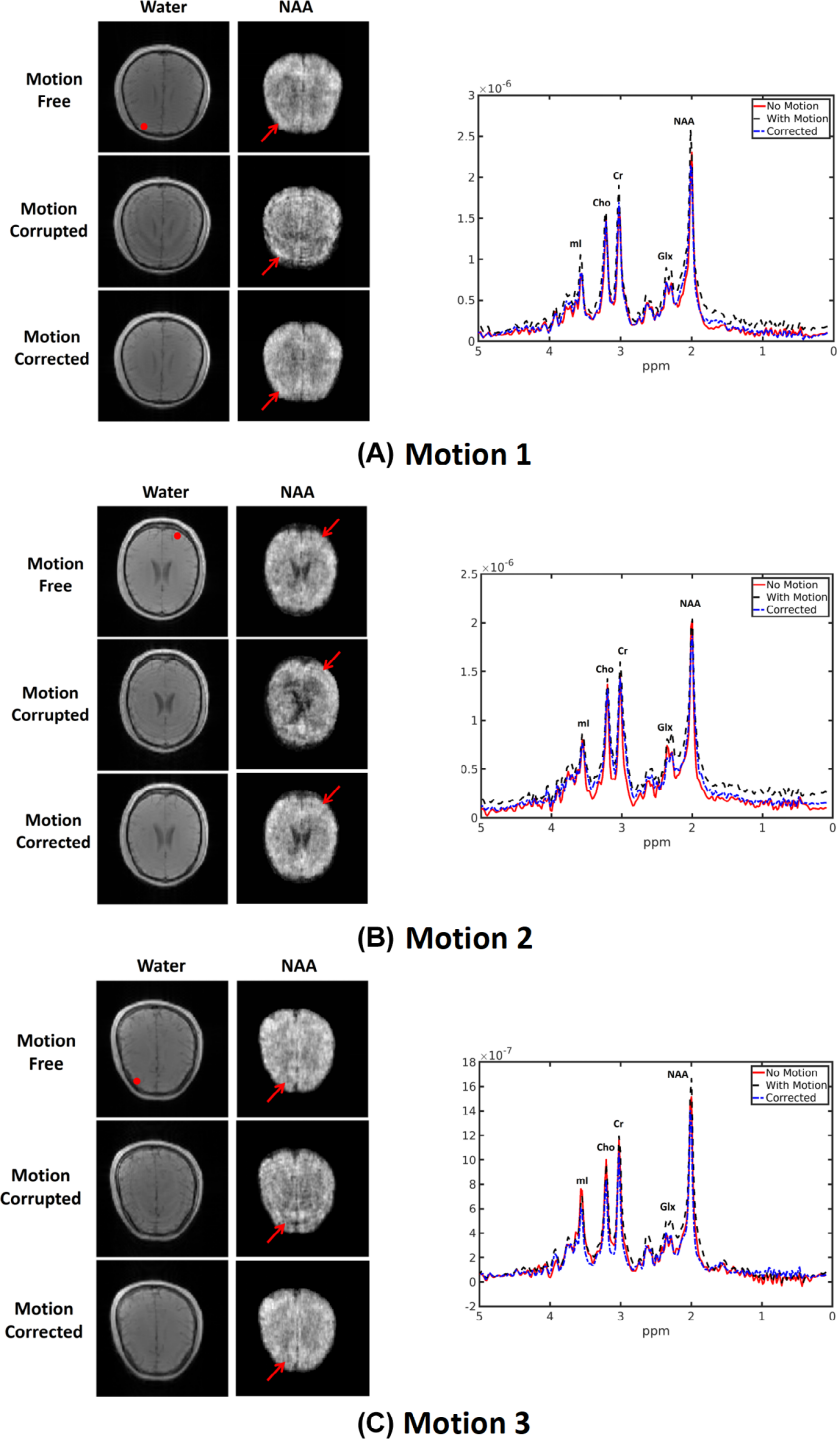
Representative comparison of MRSI results across different motion patterns, using motion‐free, motion‐corrupted, and motion‐corrected data. (A) Motion 1: brief motion returning to the original position; (B) Motion 2: brief motion without returning; (C) Motion 3: slow continuous motion. The artifacts in metabolite maps and spectral distortions in the spectra were clearly observed in the motion‐corrupted data (red arrows). These artifacts were effectively removed by the proposed method.

**FIGURE 7:**
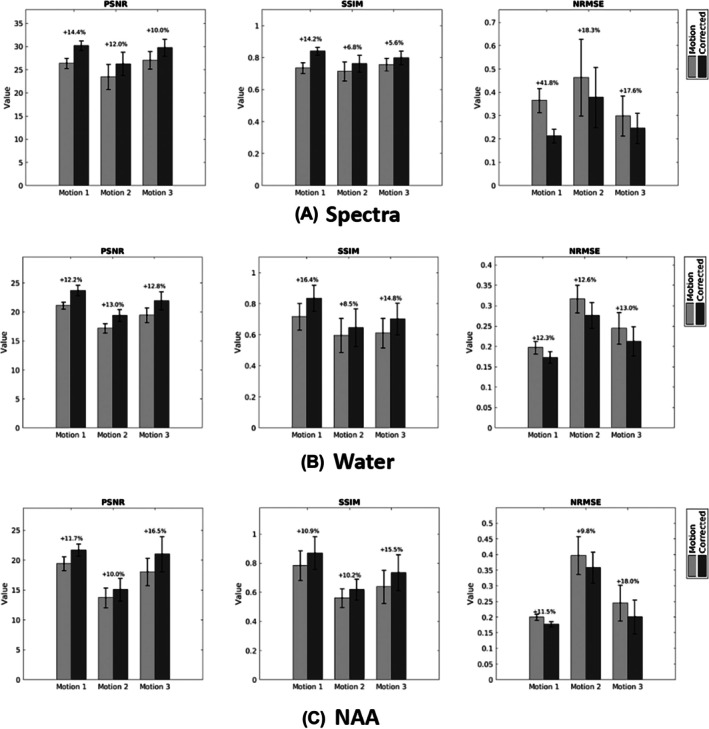
Comparison between motion‐corrupted and motion‐corrected data across three motion patterns for (A) spectra; (B) water images; and (C) NAA maps. Quantitative metrics (SSIM, NRMSE, and SNR) were performed using motion‐free data as the ground truth. The proposed correction method substantially improved consistency with the ground truth.

Figures 6, 7, and S1 illustrate the performance of the proposed motion‐correction method across different motion patterns in healthy subjects. As shown by the navigator signals in Figure S1, the estimated spatial shifts from the navigators matched our expectations for these motion patterns. Figure 6 compares the images and spectra for motion‐free, motion‐corrupted, and motion‐corrected data. As can be seen, our improved motion‐correction scheme handles these practical patterns well. Overall, our method substantially reduced motion artifacts across all motion scenarios (highlighted by red arrows). Both the water images and NAA maps demonstrated significant artifact removal, while the spectra aligned much more closely with the motion‐free reference after motion correction. In Figure 7, we evaluated the quality of images and spectra using standard metrics including PSNR, SSIM, and NRMSE, with the motion‐free data as the reference. The results demonstrate that the proposed approach achieves 10%–20% improvements in image quality across different motion scenarios.

## 4 Discussion

Our approach addresses motion correction in brain MRSI without water/lipid suppression by detecting corrupted k‐space locations using TR‐wise linear navigators and reformulating motion correction as a signal reconstruction problem. The proposed method has several advantages desirable for practical applications. First, our navigators are well‐suited for SPICE scans, leveraging key features of SPICE acquisition, such as non‐water suppression and short TR. Second, the proposed TR‐wise navigators provide very high temporal resolution for motion detection. Third, leveraging a motion‐free reference prior in the reconstruction ensures effective removal of motion artifacts from water and lipid signals, facilitating clean nuisance removal.

Acceleration is commonly used in the context of conventional water‐ and lipid‐suppressed MRSI to reduce scan time and motion artifacts [46]. However, combining our motion detection–based data rejection strategy with parallel imaging or other k‐space undersampling schemes can be challenging: the combination of sparse sampling and additional data loss from motion detection may lead to insufficient k‐space measurements for high‐quality reconstruction. This work focuses specifically on non‐water/lipid‐suppressed MRSI, where accelerated acquisition via sparse k‐space sampling is generally infeasible due to the extreme magnitude difference between water/lipid and metabolite signals (3–4 orders of magnitude). With undersampling, strong water/lipid aliasing inevitably contaminates the metabolite signals, making robust nuisance removal impractical. For this reason, existing non‐water/lipid‐suppressed MRSI methods, including the SPICE acquisition used in this study, typically acquire fully sampled k‐space and achieve acquisition efficiency through FID‐based acquisition with short TR, rather than parallel imaging or conventional undersampling [47]. Within this framework, our motion‐correction method does not introduce additional limitations on acquisition feasibility.

Although the motion observed in our experiments primarily involved rigid head movements, our method does not assume any specific motion models. Instead, it detects temporal inconsistencies in navigator signal trajectories, making it inherently sensitive to a broad range of perturbations, including pulsatile motion, venous flow, and other physiological fluctuations [48, 49]. When the perturbations, such as flow‐related signal variations, are sufficiently strong to produce noticeable navigator inconsistencies, the affected TRs can be identified and excluded from reconstruction. In our SPICE pulse sequence, we used saturation bands above and below the FOV to further reduce flow effects. In the navigator signals acquired during motion‐free scans (Figure S1a), we did not observe any obvious influence of flow, suggesting that flow effects were effectively reduced by the applied saturation bands. This suggests that flow‐related motion is not a major concern in our protocol. Future work may investigate its impact in populations with vascular abnormalities to better address flow‐related artifacts in high‐resolution MRSI.

The proposed method can be further improved to enhance its performance and clinical utility. First, motion graphs of human participants across age groups and patient populations are often more complex [50]. Although our results show that our method remains effective in many cases beyond brief motion, its performance may decrease under continuous head motion, which often results in the exclusion of a large portion of motion‐corrupted data, or under severe rotational motion, which leads to inaccurate estimation of linear phase inconsistencies. Therefore, it is not a general solution to all motions that would be encountered in practice. We hope our approach would lay a foundation for further research and development of advanced data acquisition and processing methods for motion correction in MRSI. A comprehensive evaluation with more realistic motion patterns (e.g., large portion of motion, head roll/axial rotation, and non‐rigid motion such as sneezing or swallowing) and involuntary motion experiments will be carried out in future work to fully test the performance under practical conditions. Second, the sensitivity map is currently estimated from an auxiliary FLASH scan, adding ˜20 s to the scan time. Future studies could eliminate this step using GRAPPA‐based methods [51] if central k‐space data are not affected by motion. Third, our current method implements retrospective motion correction, which requires offline correction of motion‐corrupted data. This approach has limitations, particularly when the k‐space center is corrupted (as shown in Figure S2) or when a large portion of the data is affected. Prospective correction enables real‐time handling of motion during the scan and can therefore better address severe motion. For example, Moser et al. [49] integrated volume navigators (vNav) before water suppression into a 3D FID‐MRSI sequence for real‐time motion correction and reacquisition. Compared with their vNav approach, our method uses simple linear navigators acquired at the end of each TR, requiring only a few milliseconds versus hundreds of milliseconds for vNav to cover the whole volume. This short acquisition time is particularly advantageous for FID‐MRSI, which has a relatively short TR on the order of hundreds of milliseconds, and it minimizes scan‐time increase and SAR because no additional RF pulses are applied. Although vNav can provide more accurate motion parameter estimation (both translation and rotation), our linear navigators are sufficient since our correction does not strongly depend on parameter accuracy. While our method does not provide motion parameters to update the imaging volume in real time, it is feasible in principle to extend our method for prospective correction and re‐acquisition. Using the linear navigators, we could identify motion‐corrupted TRs in real‐time and re‐acquire them when needed. This could potentially provide more flexibility in reconstruction to handle more complicated and challenging scenarios. These improvements will further enhance the clinical utility of non‐water‐suppressed MRSI.

## 5 Conclusions

This paper introduces a novel and robust motion correction technique for high‐resolution brain MRSI without water/lipid suppression. The proposed method enables efficient motion detection by inserting short navigators into each TR without extra scan time. Motion correction is achieved by data re‐generation using image reconstruction that incorporates sensitivity encoding constraints and available image priors. The effectiveness of the proposed method has been evaluated using both phantom and in vivo experimental data, which included both voluntary and involuntary motion. The results demonstrate high‐quality reconstructions, highlighting the potential of the proposed method to significantly improve the practical utility of brain MRSI without water/lipid suppression in clinical applications.

## Conflicts of Interest

Rong Guo is currently an employee of Siemens Medical Solutions USA Inc. The other authors declare no conflicts of interest.

## Supporting information


**Figure S1.** Estimated spatial shifts along three directions in the motion‐free scan and motion‐corrupted scans acquired from healthy subjects: (a) No motion; (b) Motion 1: short motion returning to the original position; (c) Motion 2: short motion without returning to the original position; (d) Motion 3: slow continuous motion. Navigator‐derived spatial shifts matched the instructed motion patterns. In Motion 1, two brief movements occurred at 73–76 s (1.22–1.26 min) and 224–227 s (3.73–3.78 min), after which the subject remained stationary; in Motion 2, brief movements at 72–76 s (1.20–1.26 min) and 223–227 s (3.71–3.78 min) did not return to the original position, producing significant spatial shifts; Motion 3 shows prolonged continuous drift. Overall, spatial shift changes are clearly observed in TRs where head movements occurred.
**Figure S2.** When motion occurs at the k‐space center, motion correction fails. The left side shows a simulated motion segment at the k‐space center, while the right side displays the reconstruction result.
