# Supplementary material for: Motion Correction in High‐Resolution 3D Brain MRSI Without Water and Lipid Suppression
Source: Magn Reson Med. 2025 Oct 10;95(3):1323–35. doi: 10.1002/mrm.70128 (PMC12746361; doi:10.1002/mrm.70128)
Supplement: Supplementary file 1 — Figure S1. Estimated spatial shifts along three directions in the motion‐free scan and motion‐corrupted scans acquired from healthy subjects: (a) No motion; (b) Motion 1: short motion returning to the original position; (c) Motion 2: short motion without returning to the original position; (d) Motion 3: slow continuous motion. Navigator‐derived spatial shifts matched the instructed motion patterns. In Motion 1, two brief movements occurred at 73–76 s (1.22–1.26 min) and 224–227 s (3.73–3.78 min), after which the subject remained stationary; in Motion 2, brief movements at 72–76 s (1.20–1.26 min) and 223–227 s (3.71–3.78 min) did not return to the original position, producing significant spatial shifts; Motion 3 shows prolonged continuous drift. Overall, spatial shift changes are clearly observed in TRs where head movements occurred. Figure S2. When motion occurs at the k‐space center, motion correction fails. The left side shows a simulated motion segment at the k‐space center, while the right side displays the reconstruction result. [file MRM-95-1323-s001.docx]

**Supporting Information**


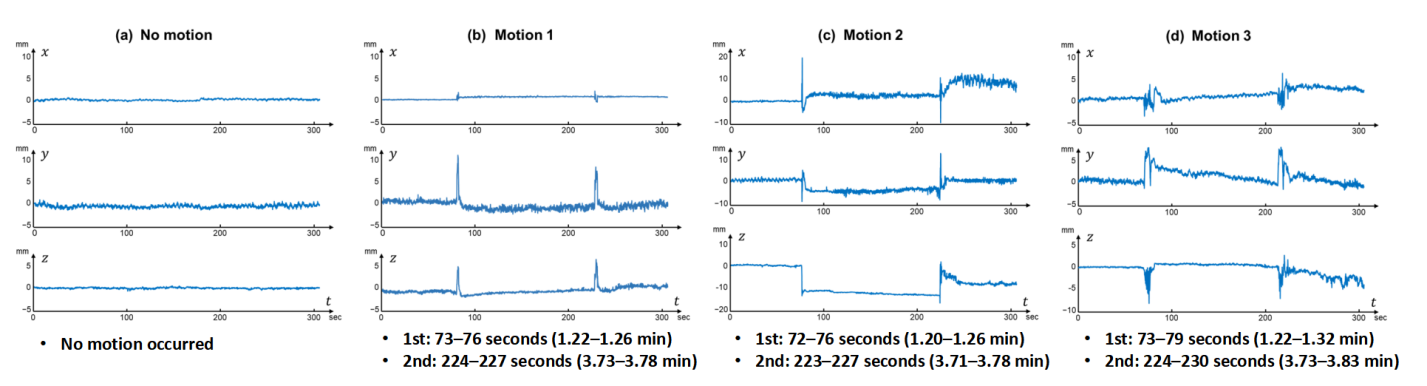


**Fig. S1**. Estimated spatial shifts along three directions in the motion-free scan and motion-corrupted scans acquired from healthy subjects: (a) No motion; (b) Motion 1: short motion returning to the original position; (c) Motion 2: short motion without returning to the original position; (d) Motion 3: slow continuous motion. Navigator-derived spatial shifts matched the instructed motion patterns. In Motion 1, two brief movements occurred at 73–76 s (1.22–1.26 min) and 224–227 s (3.73–3.78 min), after which the subject remained stationary; in Motion 2, brief movements at 72–76 s (1.20–1.26 min) and 223–227 s (3.71–3.78 min) did not return to the original position, producing significant spatial shifts; Motion 3 shows prolonged continuous drift. Overall, spatial shift changes are clearly observed in TRs where head movements occurred.


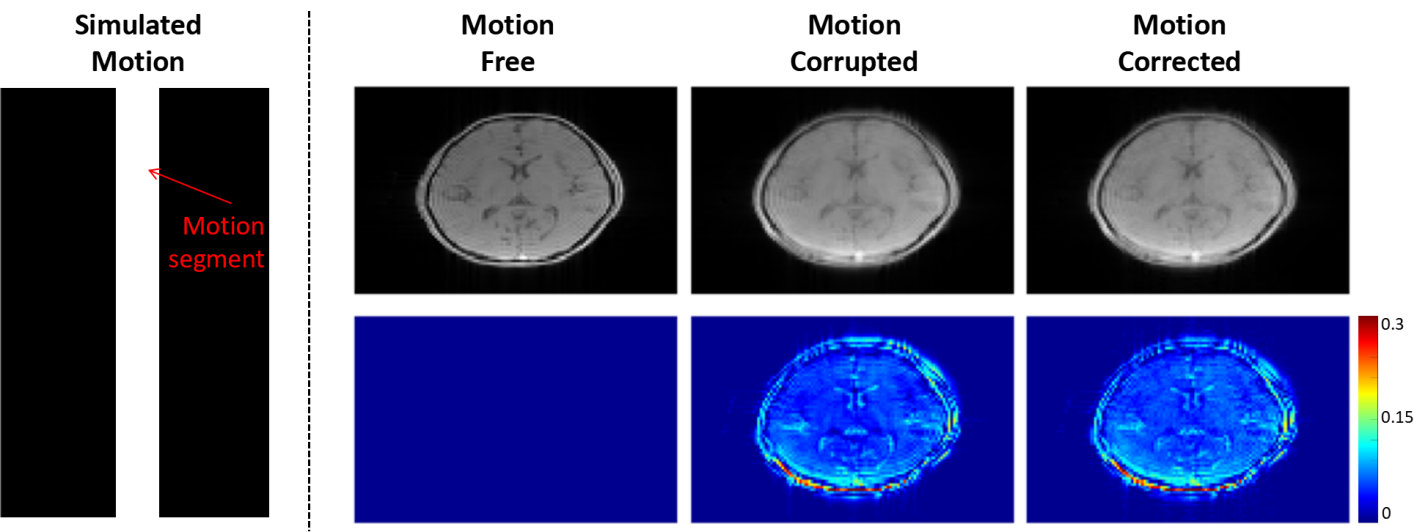


**Fig. S2**. When motion occurs at the k-space center, motion correction fails. The left side shows a simulated motion segment at the k-space center, while the right side displays the reconstruction result.
